# Editorial: The Spectrum of Lymphoid Subsets in Allergic Diseases: Immune Regulation and Immunotherapy

**DOI:** 10.3389/fimmu.2022.869781

**Published:** 2022-03-02

**Authors:** Zheng Liu, Peisong Gao, Mohamed H. Shamji

**Affiliations:** ^1^ Department of Otolaryngology-Head and Neck Surgery, Tongji Hospital, Tongji Medical College, Huazhong University of Science and Technology, Wuhan, China; ^2^ Division of Allergy and Clinical Immunology, Johns Hopkins University School of Medicine, Baltimore, MD, United States; ^3^ National Heart and Lung Institute, Imperial College London, London, United Kingdom

**Keywords:** allergy (hypersensitive anaphylaxis), lymphocyte, allergen-specific immunotherapy (AIT), regulation, biomarker

Allergic diseases, including allergic rhinitis (AR), asthma, anaphylaxis, food allergies, atopic dermatitis (AD), and drug allergies, are a group of disorders caused by hypersensitivity of the immune system to innocuous environmental antigens (1). These diseases represent a global public health problem affecting up to 50% of the world population, and the prevalence of allergic diseases has been increasing, especially in developing countries (2). While Type 2 T helper (T_H_2) cells have long been believed to play a pivotal role in allergic immune responses, growing evidence has suggested a more complex mechanism underlying the pathogenesis of allergic diseases (1). Allergen immunotherapy (AIT) is the only medical intervention that can modify the natural course of allergic diseases. To date, there are no validated biomarkers to monitor the clinical response to AIT and the mechanisms of allergy tolerance after AIT remain poorly understood (3, 4).

In recent years, the immunological mechanisms underlying allergic diseases have received increasing attention. The emerging roles of innate lymphoid cells (ILCs), T follicular helper (Tfh) cells, and B regulatory (Breg) cells in the development of allergic diseases have been identified (1, 5, 6). A better understanding of the immunological mechanisms of allergic diseases is vital to developing novel therapy and biomarkers of efficacy. Our Special Research Topic “*The Spectrum of Lymphoid Subsets in Allergic Diseases: Immune Regulation and Immunotherapy*”, compiles views from several outstanding experts in the field. Here, we discuss the main messages from four original research articles and one review article, as reported below.

Both T and B cells are believed to contribute to the pathogenesis of AD; however, the participation of novel lymphoid subsets, Tfh and Breg cells, and their interactions in childhood AD are unclear (7). Jiang et al. observed lower frequencies of CD19^+^IL-10^+^ Breg cells and higher frequencies of CD4^+^CXCR5^+^PD-1^+^ICOS^+^ Tfh cells in children with extrinsic AD than healthy controls (Jiang et al.). The frequencies of CD19^+^IL-10^+^ Breg cells correlated negatively with disease activity in children with extrinsic AD, indicating a role of Breg cells in regulating pathogenesis and disease progression of extrinsic AD (Jiang et al.). In addition, Breg cells from patients with extrinsic AD have compromised ability in inhibiting the differentiation of Tfh cells *in vitro*. This phenomenon may be associated with IL-10 deficiency in Breg cells (Jiang et al.). These observations indicate an interesting role of IL-10-producing Breg cells in Tfh cell differentiation and the pathogenesis of AD.

The discovery of ILCs has added significant insights to our views on the roles of innate immunity in allergic diseases. Growing evidence has revealed an essential role of type 2 ILCs (ILC2s) in allergic diseases. Zheng et al. summarized the common phenotypes and activation pathways of ILC2s in different allergic diseases and potential research directions to improve the understanding of their roles in different allergic diseases, hopefully leading to the development of new treatments for allergic diseases (Zheng et al.).

As a prototype of personalized medicine, effective AIT requires precise phenotyping of patients. Yet the identification of clinical accessible biomarkers is still on the way. Xie et al. reported potential biomarkers for predicting the efficacy of subcutaneous AIT (SCIT) in children with AR allergic to house dust mite (HDM) (Xie et al.). In this prospective study, children with AR were categorized into effective and ineffective group after 1-year SCIT (Xie et al.). By analyzing multiple cytokines between effective and ineffective groups, they found that serum eotaxin, interferon (IFN)-γ, interleukin (IL)-4, and macrophage migration inhibitory factor levels were potential biomarkers in predicting the response to AIT (Xie et al.). A further validation study in a cohort of 80 pediatric patients indicated that serum eotaxin and IL-4 levels were elevated in responders while IFN-γ levels decreased in responders, and serum IL-4 exhibited more reliable accuracy in predicting SCIT efficacy than eotaxin and IFN-γ (Xie et al.).

Ma et al. divided AR patients allergic to artemisia pollen into effective and ineffective groups based on the change of clinical symptoms after 1-year AIT (Ma et al.). Using liquid chromatography-tandem mass spectrometry-based proteomics, they found that thirteen proteins were changed after 1-year therapy in effective group but remained unchanged in ineffective groups (Ma et al.). Four proteins [mucin 5B, lipopolysaccharide-binding protein, C4b-binding protein beta chain, and leukotriene A-4 hydrolase (LTA4H)] were further identified as potential biomarkers for the efficacy of AIT based on their association with allergy and the protein fold change between AIT responders and non-responders (Ma et al.). LTA4H effectively distinguished responders from the non-responders (AUC = 0.844), suggesting that serum LTA4H might be a potential biomarker for predicting the efficiency of AIT (Ma et al.).

Yang et al. investigated the persistence and evolution of HDM-specific IgE and IgG4 and explored their correlation with clinical responses during AIT in AR with/without asthma patients (Yang et al.). They observed that sIgG4 to *Dermatophagoides pteronyssinus* (Der p) 1, *Dermatophagoides farina* (Der f) 1, Der p 2, Der f 2, and Der p 21 significantly increased at 18-month after AIT compared to the baseline; however, sIgE to HDM components demonstrated no difference at baseline and 18-month in the AIT group (Yang et al.). In addition, the changes of sIgE, sIgG4, sIgE/sIgG4 ratio and the numbers of positive HDM components had no correlation with the improvement of symptoms after AIT (Yang et al.). They concluded that although AIT robustly induced the production of sIgG4 to HDM components, their changes were not qualified as a biomarker to evaluate the efficacy of AIT (Yang et al.). These three papers added new knowledge regarding the biomarkers for patient selection and efficacy monitoring in AIT. However, all of them are limited by the small sample size and lack of external and multicenter validation. These findings need to be confirmed in future investigations before their application in clinic settings.

Despite all the progress made in the last decades, we are still at an early stage in our understanding of the pathogenic of allergic diseases. The intricate interactions between T cell, B cell, and ILCs and their roles in allergic diseases still need to be further clarified. Increased knowledge of the novel role of lymphoid subsets in the development of allergic diseases and tolerance induction during AIT and the biomarkers to monitor or predict the efficacy of AIT are encouraging, and hopefully, it will continue to encourage more studies to reinstate the balance of these cells for the prevention and treatment of allergic diseases.

## Author Contributions

All authors contributed to the article and approved the submitted version.

## Funding

This study was supported by the National Natural Science Foundation of China (NSFC) grants 8192010801, 82130030 and 81630024 (ZL) and the US National Institutes of Health (NIH) grants R01AI153331 and R01AI141642 (PG).

## Conflict of Interest

The authors declare that the research was conducted in the absence of any commercial or financial relationships that could be construed as a potential conflict of interest.

## Publisher’s Note

All claims expressed in this article are solely those of the authors and do not necessarily represent those of their affiliated organizations, or those of the publisher, the editors and the reviewers. Any product that may be evaluated in this article, or claim that may be made by its manufacturer, is not guaranteed or endorsed by the publisher.
